# Perspectives and Needs of Malaysian Patients With Diabetes for a Mobile Health App Support on Self-Management of Diabetes: Qualitative Study

**DOI:** 10.2196/40968

**Published:** 2023-10-23

**Authors:** Wei Thing Sze, Suk Guan Kow

**Affiliations:** 1 Faculty of Pharmacy SEGi University Selangor Malaysia; 2 Department of Biomedical Informatics The University of Tokyo Tokyo Japan

**Keywords:** mHealth, self-management, diabetes, remote monitoring, telehealth, telemedicine

## Abstract

**Background:**

Effective self-management of diabetes is crucial for improving clinical outcomes by maintaining glucose levels and preventing the exacerbation of the condition. Mobile health (mHealth) has demonstrated its significance in enhancing self-management practices. However, only 20% of Malaysians are familiar with mHealth technologies and use them for health management.

**Objective:**

This study aims to explore the perceived benefits and challenges, needs and preferences, and willingness of patients with diabetes to use mHealth apps for self-management of diabetes.

**Methods:**

The study involved one-on-one semistructured online interviews with a total of 15 participants, all of whom were aged 18 years or older and had been diagnosed with diabetes for more than 6 months. An interview guide was developed based on the constructs of the Technology Acceptance Model (TAM), the Health Information Technology Acceptance Model (HITAM), and the aesthetics factor derived from the Mobile Application Rating Scale. All interviews were recorded in audio format and transcribed verbatim. The interview content was then organized and coded using ATLAS.ti version 8. Thematic analysis was conducted in accordance with the recommended guidelines for analyzing the data.

**Results:**

From the interviews with participants, 3 key themes emerged regarding the perceived benefits of using mHealth app support in diabetes self-management. These themes were the ability to track and monitor diabetes control, assistance in making lifestyle modifications, and the facilitation of more informed treatment decision-making for health care professionals. The interviews with participants revealed 4 prominent themes regarding the perceived barriers to using mHealth app support for diabetes self-management. These themes were a lack of awareness about the availability of mHealth support, insufficient support in using mHealth apps, the perception that current mHealth apps do not align with users’ specific needs, and limited digital literacy among users. The interviews with participants unveiled 4 key themes related to their needs and preferences concerning mHealth app support for diabetes self-management. These themes were the desire for educational information, user-friendly design features, carbohydrate-counting functionality, and the ability to engage socially with both peers and health care professionals. The majority of participants expressed their willingness to use mHealth apps if they received recommendations and guidance from health care professionals.

**Conclusions:**

Patients generally perceive mHealth app support as beneficial for diabetes self-management and are willing to use these apps, particularly if recommended by health care professionals. However, several barriers may hinder the utilization of mHealth apps, including a lack of awareness and recommendations regarding these apps from health care professionals. To ensure the effective development of mHealth app support systems for diabetes self-management, it is crucial to implement user-centered design processes that consider the specific needs and preferences of patients. This approach will help create apps that are tailored to the requirements of individuals managing diabetes.

## Introduction

### Background

Diabetes is rapidly becoming one of the most prevalent diseases worldwide in the 21st century. Approximately 537 million people across the globe are affected by diabetes, with an estimated 6.7 million deaths directly linked to the condition in 2021 [1]. In Malaysia, the National Diabetes Registry recorded the enrollment of nearly 1.7 million patients, with 902,991 actively managed diabetes cases reported at the close of 2020. The majority of these patients were diagnosed with type 2 diabetes mellitus (T2DM) at 99.33%, followed by type 1 diabetes mellitus at 0.59%, and other types at 0.06%. Among the ethnic groups, the Malay community had the highest prevalence of diabetes, followed by the Chinese, Indians, and other ethnic groups [2]. T2DM is recognized as the most prevalent form of diabetes and has emerged as a significant public health issue in Malaysia. The rising prevalence of T2DM can be attributed to various factors, including unhealthy dietary habits, excessive carbohydrate intake, and a lack of physical activity [3]. Patients with inadequate diabetes management face an elevated risk of both mortality and morbidity. The chronic complications linked to diabetes, including neuropathy, retinopathy, cardiovascular disease, stroke, and the necessity for foot amputations, can substantially diminish patients’ quality of life [4].

To prevent the worsening of the disease, diabetes self-management is crucial for individuals with diabetes. Patients are encouraged to make lifestyle changes and adopt healthier habits to maintain better control over their blood glucose levels [5]. In recent years, there has been rapid development in products and services aimed at self-care for diabetes [6]. Digital health tools have evolved to facilitate disease management, offering personalized functions for self-management and enabling communication between patients and health care professionals [7,8]. Wearable devices and mobile apps have demonstrated their ability to enhance patients’ blood glucose levels, encourage self-management behaviors, improve medication adherence, and increase clinical satisfaction [9,10].

### Self-Management Among Patients With Diabetes

Diabetes self-management is strongly encouraged among patients with diabetes as it has the potential to lower hemoglobin A_1c_ (HbA_1c_) levels, consequently reducing the risk of exacerbations and long-term complications [11-13]. It has the potential to alleviate the burden on health care providers by promoting self-monitoring at home among patients [14,15]. Self-management behaviors encompass glucose monitoring, maintaining a healthy diet, engaging in regular physical exercise, adhering to medication regimens, risk reduction, developing coping skills, and problem-solving [16]. However, the practice of self-care among patients with diabetes in Malaysia remains relatively low. It has been reported that many patients are noncompliant with medication adherence, physically inactive, and have unhealthy eating habits, all of which contribute to the deterioration of their blood glucose levels [17]. Potential barriers to self-care practices are a lack of knowledge and skills in diabetes self-management, insufficient counseling, a low perception of the severity of the disease, a lack of motivation and support, and financial constraints [18-20].

### Mobile Health Support in Self-Management

Mobile health (mHealth) is defined as “the use of mobile and wireless technologies to support the achievement of health objectives” [21]. mHealth technologies are typically patient-facing and are available on patients’ mobile devices. Some of the mHealth devices are smartphones, wearable activity trackers, wireless-connected scales, blood pressure cuffs, pulse oximeters, and glucometers. Patients have been able to gain a deeper understanding of their health condition and make adjustments to their lifestyle habits through the use of mHealth [22]. mHealth aids in various aspects of self-management by collecting user health data and offering personalized information, instructions, graphical representations, guidance, and reminders [23]. However, it has been reported that only 20% of Malaysians are familiar with mHealth and actively use it [24]. The limited adoption of mHealth can be attributed to various factors, including usability concerns, perceived complexity in usage, and the absence of integration with electronic health records [25,26]. Additional factors contributing to this situation are cost-related concerns, internet connectivity issues, a lack of knowledge and skills, the perception of the limited usefulness of digital devices, and concerns about data security. These factors can potentially influence patients’ attitudes and behaviors regarding digital health interventions [27].

### Patients’ Perspectives on Using mHealth Support for Disease Management

Numerous studies have explored patients’ perspectives on using mHealth support for disease management. Some of these studies have indicated that most patients, particularly the younger generation and frequent computer users, express a strong interest in using mHealth for disease management [28-30]. Patients have described the ease of communication with clinicians, enhanced comprehension of their disease conditions, fewer frequent hospital visits, and an overall improvement in wellness and quality of life as benefits resulting from the use of mHealth [31-33]. However, older individuals have expressed their lack of familiarity with technology operations and a preference for traditional methods involving manual recording of results [28]. Additionally, they, like others, have raised concerns regarding data privacy, challenges in accessing data, the absence of personalized features, and financial constraints, all of which have been highlighted in previous studies [28,34].

### Justification for Research

Previous research has examined the views and perceptions of Malaysian patients regarding general mHealth support for various health conditions [24,35], but there has been limited focus on its specific usage among patients with diabetes. Moreover, there is a dearth of knowledge concerning patients’ needs and preferences for mHealth interventions, as previous studies in Malaysia have primarily concentrated on patients’ perceptions and experiences with mHealth [24,28,36]. Recent research has revealed that as many as 80% of participants in mHealth interventions engage at only a minimal level [37], and approximately one-quarter of downloaded health apps are used just once [38]. Hence, researchers must delve into how the intervention can align with users’ needs and preferences by gaining a profound qualitative understanding of the target population. A comprehensive understanding of patients’ needs and preferences will enable developers to design mHealth features that are not only usable but also engaging [39]. This step aligns with the “empathize with the target users” phase in the design thinking process and frequently serves as the initial stage in the development of mHealth interventions [40].

### Study Objectives

This study aims to investigate the perceived benefits and barriers of using mHealth apps among Malaysian patients with diabetes. The study also aims to uncover their specific needs and preferences for mHealth apps in the context of self-managing diabetes. Furthermore, we sought to determine their willingness to embrace mHealth apps as a tool for diabetes self-management.

## Methods

### Study Design and Recruitment of Participants

This research utilized an exploratory, qualitative design using a phenomenological approach. In-depth, one-on-one, semistructured qualitative interviews were conducted between September 2021 and November 2021. The study adhered to the guidelines outlined in the COREQ (Consolidated Criteria for Reporting Qualitative Research; Multimedia Appendix 1) checklist in both its design and reporting. Participants were recruited via advertisements posted on social media platforms. We used purposive sampling, specifically utilizing the maximum variation sampling method, to ensure a diverse representation of patients across various demographics, including age, gender, ethnicity, income level, and educational background, to capture a wide range of perspectives. Because of challenges in recruiting patients of Indian ethnicity, we applied a snowball sampling method to reach this population. This involved leveraging the social networks of existing participants, who recommended potential participants for the study. Inclusion criteria for participation were as follows: individuals diagnosed with diabetes for more than 6 months, aged 18 years or older, proficient in English, and capable of accessing the internet and using the Zoom web conferencing tool (Zoom Video Communications, Inc.). Participants were provided with a comprehensive briefing to address any concerns or queries they may have had regarding the study. An information sheet was provided to participants, and their formal consent to participate in the study was obtained. Additionally, participants were offered a 10 Malaysian Ringgit (US $2.12) e-wallet gift token as compensation for their time.

### Positionality of the Research Team

WTS, a pharmacist with extensive experience in delivering medication education and counseling to patients with diabetes from various Malaysian ethnic backgrounds, brought valuable insights into the study. Her wealth of experience enabled her to empathize with the experiences and challenges encountered by patients with diabetes, which in turn influenced her perspectives during the data analysis phase. It is important to note that WTS did not conduct the interviews during the data collection process but played a significant role in the interpretive analysis of the interview transcripts.

SGK’s positionality as a trainee pharmacist, coupled with her direct experiences of engaging with patients with diabetes from diverse cultural backgrounds, enabled her to establish a connection with the patients’ perspectives and requirements in diabetes self-management. In her role as an interviewer, SGK established a personal rapport with the participants and effectively empathized with their lived experiences. This connection was instrumental in shaping her engagement with the data during the analysis process.

### Interview Guide

We developed a semistructured interview guide (Multimedia Appendix 2), drawing inspiration from Anderson et al [11]. The interview questions were rooted in the constructs of “Perceived ease of Use” and “Perceived Usefulness” from the Technology Acceptance Model (TAM) [41], personal and social factors as outlined in the Health Information Technology Acceptance Model (HITAM) [42], and the aesthetics factor derived from the Mobile Application Rating Scale (MARS) [43]. TAM evaluates a user’s attitude toward adopting a technology, comprising Perceived Usefulness and Perceived Ease of Use. HITAM, by contrast, extends the TAM concepts by incorporating the Health Belief Model [42]. HITAM is designed to describe the attitude and behavioral intentions of health consumers when they encounter health information technology. HITAM is particularly well-suited for our research, given its comprehensive consideration of various facets of health behaviors. It encompasses behavioral, personal, social, and information technology factors, including health status, health beliefs and concerns, subjective norms (social pressure within the diabetes community), health information technology reliability (the demonstrability of results through direct experience with the technology or information gathered from other consumers), and health information technology self-efficacy (confidence in using mHealth). Conversely, MARS evaluates app quality based on 4 constructs: engagement, aesthetics, functionality, and the quality of information [43]. As the quality of apps significantly impacts the user experience, we incorporated these MARS constructs into our interview guide.

We also incorporated additional questions into the interview guide to delve into participants’ acceptance factors regarding the use of digital health tools (eg, “What form of information would you find most useful?”) [11]. This question yielded comprehensive insights into participants’ needs and preferences regarding mHealth support. We conducted a pilot test to assess the suitability of the interview questions and to help the study researcher become proficient in guiding the conversation effectively.

### Data Collection

One-on-one semistructured interviews were conducted by author SGK using Zoom. No prior relationship existed between the interviewer and participants before the interviews. Demographic information, including age, gender, ethnicity, type of diabetes, duration of diabetes, employment status, and educational level, was collected. Before the interviews, participants were shown a 5-minute video presentation explaining the use of mHealth apps in diabetes. This presentation aimed to ensure that participants had a clear understanding of the concepts and ideas related to the use of mHealth apps for self-managing diabetes. During the interviews, information regarding patients’ perceived benefits and barriers, as well as their needs and preferences for using mHealth app support in diabetes self-management, was explored in accordance with the interview guide. The interviews involved asking open-ended questions, with the interviewer also posing follow-up and probing questions to encourage participants to provide comprehensive responses. On average, the interviews lasted approximately 45 minutes. All interviews were recorded in audio format and subsequently transcribed verbatim by the researcher immediately after each interview. The interviewer also took field notes during and immediately after the interviews. We did not discover any new, pertinent codes or themes emerging from the interview transcript of participant 13. Consequently, we decided to conclude the recruitment process after collecting data from a total of 15 interviews [44,45].

### Data Analysis

The interview content was systematically organized and coded using ATLAS.ti (ATLAS.ti Scientific Software Development GmbH), version 8. The data were then subjected to thematic analysis, following the guidelines outlined by Braun and Clarke [46]. Both WTS and SGK independently coded all the transcripts using NVivo software (QSR International). Initially, they read the transcripts multiple times to become thoroughly acquainted with the data. Subsequently, they independently coded the transcripts, creating initial codes based on meaningful paragraphs and grouping them under potential themes if they shared similar contexts. The coders engaged in coding meetings to discuss the codes and resolve any discrepancies that arose during the coding process. The codebook underwent refinements through iterative reviews of new codes and themes derived from additional interviews. Ultimately, the final set of themes was determined through consensus among all the researchers. Transcripts were not returned to the participants for comments or corrections, nor were participants asked to provide feedback on the findings.

### Ethical Considerations

This study received approval from the SEGi University Research Ethics Committee (approval number SEGIEC/SR/FOP/29/2021-2022). Participant confidentiality was upheld through the use of study codes. Participation in the study was entirely voluntary, and participants were informed that they had the option to withdraw at any point. Data access was strictly limited to the study researchers. The ethics procedures of this study adhere to the principles outlined in the Declaration of Helsinki.

## Results

### Demographics of Participants

A total of 15 participants were recruited for the study. Detailed demographic characteristics of the participants are presented in Table 1. Notably, less than half of the participants (6/15, 40%) were using digital health support for diabetes self-management.

**Table 1:** Participant characteristics.

Participant characteristics		Values (N=15), n (%)
**Use of a mobile health app for self-management**		
	Yes	6 (40)
	No	9 (60)
**Age (years)**		
	18-30	4 (27)
	31-40	2 (13)
	41-50	1 (7)
	51-60	3 (20)
	61-65	0 (0)
	>65	5 (33)
**Gender**		
	Female	8 (53)
	Male	7 (47)
**Race**		
	Chinese	6 (40)
	Indian	4 (27)
	Malay	5 (33)
**Diabetes type**		
	Type 1	3 (20)
	Type 2	12 (80)
**Duration of diabetes**		
	6 months-2 years	1 (7)
	2-5 years	4 (27)
	>5 years	10 (67)
**Working status**		
	Employed	9 (60)
	Unemployed	6 (40)
**Education level**		
	Primary education	0 (0)
	Secondary education	7 (47)
	Tertiary education	8 (53)
**Diabetes management**		
	Lifestyle modification only	3 (20)
	Oral medication only	8 (53)
	Oral medication and insulin	1 (7)
	Insulin only	3 (20)

### Perceived Benefits of Using mHealth App Support

#### Overview

**Figure 1:**
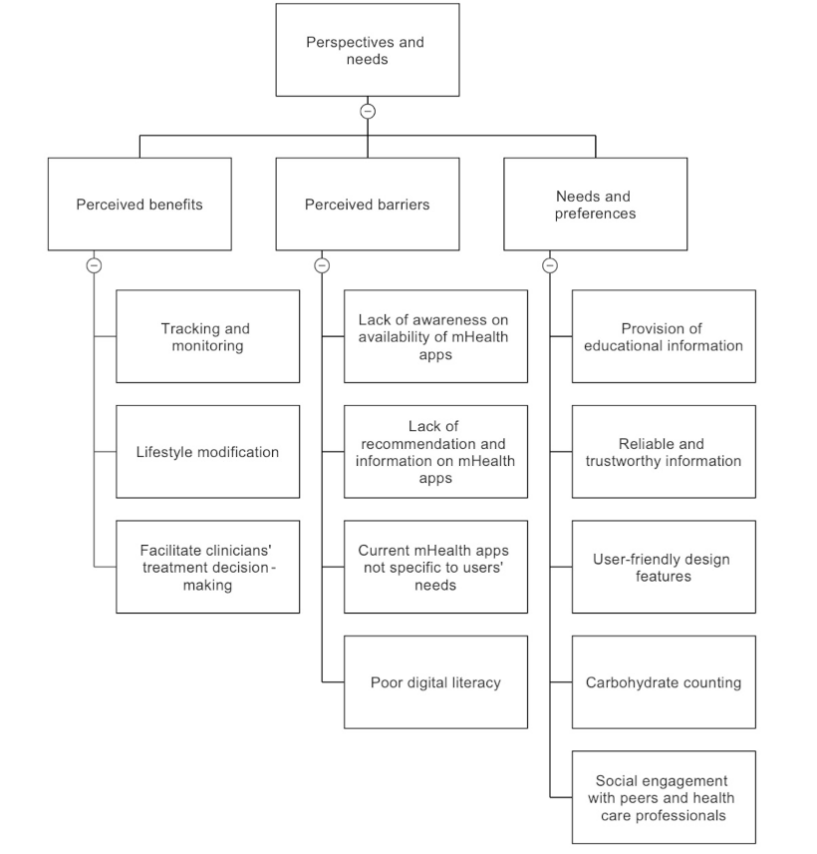
Generated themes on the perspectives and needs of Malaysian patients with diabetes on mobile health (mHealth) apps for diabetes self-management.

The interview with participants revealed 3 key themes regarding the perceived benefits of using mHealth app support in diabetes self-management: tracking and monitoring diabetes control, aiding in lifestyle modification, and facilitating improved treatment decision-making for health care providers (Figure 1).

#### Theme 1: Tracking and Monitoring Diabetes Control

The majority of participants believed that using an mHealth app could assist them in tracking and monitoring their diabetes control effectively. They were confident that they could effortlessly record and monitor their health data, allowing them to revisit previous records and observe their health trends at their convenience.

I like it when there’s a wealth of data there, you can kind of look back and see what's your average? What is your trend? What are your habits?

Participant 1, Female, age 18-30 years, mHealth app user

#### Theme 2: Assist in Lifestyle Modification

The support provided by mHealth apps can play a significant role in facilitating lifestyle modifications for individuals with diabetes. A majority of participants expressed the belief that they could establish dietary goals and targets by monitoring their blood glucose trends using these apps. Additionally, participants felt that they could practice diabetes self-management independently, without the need for external reminders or assistance.

When I view the data on the app, I want them to reach a certain level or goal.

Participant 1, Female, age 18-30 years, mHealth app user

I cannot depend on other people to control my diabetes or to remind me of what to do every day. The app can help me build a habit, like a new lifestyle.

Participant 9, Male, age 51-60 years, mHealth app nonuser

When my blood sugar level is high from the app, I will start to do something...Next day I will cut down a lot of carbs and then do more exercises.

Participant 11, Female, age 65 years and above, mHealth app nonuser

#### Theme 3: Facilitate Better Treatment Decision-Making for Clinicians

Participants were confident that mHealth apps could assist health care professionals in making more informed decisions about their treatment plans. They could readily share and discuss their health records with health care providers, thanks to the data stored in these apps.

By checking the data with our doctor, we can discuss with our doctor on the trend.

Participant 4, male, age 18-30 years, mHealth app nonuser

With more data, I think doctors can make better treatment decisions.

Participant 7, Female, age 18-30 years, mHealth app user

### Barriers and Challenges to the Adoption of mHealth Support

#### Overview

During the interviews with participants, 4 distinct themes surfaced concerning the perceived barriers to using mHealth support for diabetes self-management. These included a lack of awareness regarding the availability of mHealth support, insufficient support in the usage of mHealth technology, the inadequacy of current mHealth apps to cater to users’ specific needs, and limited digital literacy.

#### Theme 1: Lack of Awareness on the Availability of mHealth Apps

Our findings indicated that a significant portion of non–mHealth users were unaware of the availability of mHealth app support for diabetes management. Participants expressed that they had no prior knowledge about the existence of mHealth apps designed for diabetes self-management, and as a result, had not considered using such apps for managing their diabetes.

I don’t know that diabetes apps exist.

Participant 5, Male, age above 65 years, mHealth app nonuser

Well, in the first place nobody has recommended me. This is the first time I hear of it, I have very little knowledge of this actually.

Participant 14, Female, age 18-30 years, mHealth app nonuser

#### Theme 2: Lack of Recommendation and Information on Using mHealth Apps

Conversely, participants also conveyed that there was a noticeable absence of recommendations and information regarding the use of mHealth apps. They reported that health care professionals, primarily doctors, provided guidance on medications and lifestyle modifications but did not suggest or endorse any mHealth apps or other digital health resources for managing diabetes.

No the doctor didn’t mention it, so far no.

Participant 5, Male, age above 65 years, mHealth app nonuser

Nobody has informed me, not even the clinic that I go to. They have not informed me anything.

Participant 11, Female, age 65 years and above, mHealth app nonuser

#### Theme 3: Current mHealth Apps Not Specific to Users’ Needs

Several participants had negative experiences with mHealth apps, primarily due to the perception that these apps did not cater specifically to their individual needs. They described the current apps as having limited options and functionalities for personalized diabetes self-management. These limitations encompassed the absence of certain critical information, the requirement for users to manually input data, and diet recommendations in the apps that were not tailored to the local context.

I remembered that some apps have carbohydrates counting, but they are not very specific yet, for example, for rice, only brown rice and white are listed on the app.

Participant 15, Female, age 51-60 years, mHealth app user

We can set the sugar intake limit or a range that we want...But the application did not specify the limit for pregnant or normal patients. We need to set the limit ourselves.

Participant 12, Female, age 31-40 years, mHealth app user

#### Theme 4: Poor Digital Literacy

Furthermore, limited digital literacy emerged as a significant barrier to the adoption of mHealth apps, particularly among the older generation. Older participants expressed that they were not accustomed to using digital technology, leading to difficulties in operating newer technologies such as mHealth apps. They mentioned that they lacked the knowledge and skills required to use mHealth apps and often preferred using basic mobile phones instead.

I am not computer user, so I prefer the simpler method.

Participant 8, Male, age above 65 years, mHealth app nonuser

I'm an old school I’m not used to tech stuffs.

Participant 9, Male, age 51-60 years, mHealth app nonuser

I use the normal phone, I didn’t use smartphone.

Participant 2, Female, age 51-60 years, mHealth app nonuser

### Needs and Preferences Toward mHealth App Support

#### Overview

During the interviews with participants, 4 distinct themes emerged concerning their needs and preferences regarding mHealth app support for diabetes self-management. These included the desire for educational information, user-friendly design features, features related to carbohydrate counting, and the opportunity for social engagement with peers and health care professionals.

#### Theme 1: Provision of Educational Information

Participants offered several suggestions to enhance the design of digital health solutions aimed at facilitating diabetes self-management. Primarily, they expressed a preference for mHealth apps to include educational content, particularly information related to diet, glucose control, and hypoglycemia management. Furthermore, participants emphasized that it would be beneficial if these apps offered dietary advice, specifying which foods to include and avoid while allowing users to set targets for achieving desired glucose levels. Additionally, they suggested the inclusion of Asian recipes within the apps alongside Western recipes, ensuring a more culturally relevant and diverse selection.

I prefer the apps to offer education, such as what type of food is better and what type of food I should avoid...

Participant 6, Male, age 65 years and above, mHealth app nonuser

I prefer to know the range of healthy blood glucose level, and then maybe some info on the hypoglycaemia management.

Participant 12, Female, age 31-40 years, mHealth app user

A lot of the apps for diabetes have recipes, but they are mostly Western recipes. If they have Malaysian or Asian types of recipes, that would be good.

Participant 13, Female, age 31-40 years, mHealth app user

Nevertheless, participants also reported that repetitive educational messages are less desirable.

Sometimes the apps keep on sending the same thing (the educational text messages). I mean these things are all very similar. It's just like maybe I read them (the educational text message) before, so they are no more interesting to me.

Participant 3, Male, age 41-50 years, mHealth apps user

#### Theme 2: User-Friendly Design Features

Some participants expressed the desire for different digital health devices and apps to be able to interoperate and communicate with each other seamlessly. They also emphasized the importance of personalized functions within the apps, including options to access information through videos, articles, or by sharing information and engaging with peers in a forum-style environment.

If these devices can communicate with each other, then it's perfect...

Participant 7, Female, age 18-30 years, mHealth app user

I prefer reading the article and see the video, because I don't like to see pictures...

Participant 10, Male, age 65 years and above, mHealth app nonuser

Participants also expressed a desire for reminder and notification features within mHealth apps. They were interested in using apps that could assist them in monitoring their health and provide alerts or notifications when their health was not well-controlled. Additionally, they highlighted the need for medication reminders, particularly for those who were managing multiple medications and had busy schedules.

It will be good when I have low blood sugar, a message pop up to inform me something, like whether I have certain symptoms...Like a notification reminder...

Participant 1, Female, 18-30 years, mHealth app user

...for patients who take a lot of medication, reminders for them will be really good.

Participant 4, male, age 18-30 years, mHealth app nonuser

#### Theme 3: Carbohydrate Counting

Furthermore, participants recommended the inclusion of a carbohydrate-counting feature in mHealth apps. Many participants emphasized the importance of being able to monitor their food intake and track their diet as a crucial aspect of diabetes self-management.

If we can put a picture of our meal in the app...which can scan how much carbohydrate is there through the picture...that would be a very big bonus point for all of us.

Participant 4, male, age 65 years and above, mHealth app nonuser

I would like to know what kind of food (and they) contains how much carbs.

Participant 9, Male, age 51-60 years, mHealth app nonuser

#### Theme 4: Social Engagement With Peers and Health Care Professionals

Finally, participants expressed a preference for mHealth apps to include engaging features that facilitate effective communication between health care professionals and other patients. In their daily lives, participants often sought advice and shared experiences with their friends with diabetes when facing challenges in diabetes management. Additionally, some participants found it challenging to consult with doctors in between appointments. Therefore, participants suggested incorporating more opportunities for interaction with fellow patients with diabetes and health care professionals through the mHealth apps to address these needs and enhance their diabetes self-management experience.

...with friends that also have diabetes, we also discuss among ourselves how to go about improving diabetes.

Participant 5, Male, age 65 years and above, mHealth app nonuser

...the limitation for me is that we only see the doctor every three months. So in between if whatever happened, it is hard to ask my doctor.

Participant 7, Female, age 18-30 years, mHealth app user

### Willingness to Use mHealth Support for Diabetes Self-Management

Currently, less than half of the participants are using mHealth apps to support their diabetes management. However, most participants expressed a strong willingness to embrace mHealth app support in the future. The majority of participants indicated their readiness to use mHealth app support provided they received appropriate guidance and recommendations. Furthermore, participants exhibited a positive attitude toward using mHealth apps, especially when these apps proved to be beneficial in managing their diabetes conditions.

I wouldn’t mind to use the app if it can help to manage my diabetes level.

Participant 6, Male, age above 65 years, mHealth app nonuser

I think it will be a very good support.

Participant 8, Male, age above 65 years, mHealth app nonuser

Yeah, I don’t mind doing that, if there is somebody to recommend or introduce to me.

Participant 11, Female, age above 65 years, mHealth app nonuser

## Discussion

### Perceived Benefits of Using Digital Health Support

In general, participants held a positive perception of mHealth app interventions, considering them as beneficial for enhancing their diabetes management. This viewpoint aligns with existing evidence that suggests an association between the utilization of digital health support and the potential improvement in diabetes self-management among individuals with diabetes [47]. Patients can acquire a more comprehensive understanding of how various factors impact their blood glucose levels by tracking their data and visualizing trends through mHealth apps [48]. Additionally, these apps facilitate easy recording of blood glucose readings compared with traditional paper-based methods [49]. It is worth noting that frequent monitoring of blood glucose trends is recognized as beneficial for achieving better glycemic control [25].

In this study, we observed that patients expressed enthusiasm for using mHealth apps due to their potential to support lifestyle modification. This aligns with findings from a study conducted by Fleming et al [50], who reported that patients were more motivated to engage in glucose monitoring and make lifestyle modifications when using digital health support. From the patients’ standpoint, mHealth apps enabled them to share their health data with health care professionals. The utilization of mHealth apps can support health care professionals in making more informed clinical decisions by providing access to patient data. Studies have shown that the use of mobile apps for clinical decision support can lead to improvements in the appropriateness of diagnoses and clinical outcomes [51].

### Barriers and Challenges to the Adoption of mHealth

Patients’ perceived barriers and challenges related to the adoption of mHealth apps primarily center on the limited options and functionalities offered by the apps, as well as issues with app usability. This includes the inconvenience of having to manually input health data into the app. An assessment of free Android health care apps unveiled that the majority of mHealth apps only supported manual data entry, highlighting the prevalent need for users to input their health information manually [52]. Fu et al [53] proposed the enhancement of user satisfaction by integrating Health Behavior Theory into the design and development of digital health technology. Additionally, the concern about poor digital literacy was particularly notable among older participants. The older generations frequently voiced challenges related to navigating mHealth apps and generally reported experiencing fewer benefits from mobile app usage compared with younger generations [14,54]. Developers should exercise special care when designing mHealth apps for older individuals. Considerations should include factors such as font size, color contrast, button visibility, and the inclusion of helpful tips and explanations to ensure that these apps are accessible and user-friendly for older users [55].

There was a clear lack of awareness among patients regarding the availability of mHealth support. Most patients had never received guidance from health care professionals on the use of mHealth apps for diabetes self-management. Research has shown that the absence of patient-health professional interactions is a significant barrier to the adoption of mHealth technology. A study by Biruk and Abetu [56] reported limited knowledge about digital health support for disease management among health care professionals in North West Ethiopia. Therefore, it is essential to educate health care professionals about the advantages of incorporating mHealth interventions into the management of chronic diseases as a first step.

### Patients’ Needs and Preferences Toward Digital Health Support

Our study also highlights opportunities for improvement in the design and functionality of mHealth apps for diabetes management. One of the key recommendations that emerged from the findings was the need for the provision of educational information within these apps. Studies have demonstrated that numerous mHealth apps do not offer sufficient educational information to users [25]. It is worth noting that education provided within mHealth apps can enhance self-care practices [57]. Additionally, patients frequently encountered difficulties in calculating the carbohydrate content of their meals and expressed a preference for apps to include a feature that allows them to scan images for calorie information. This is particularly significant because carbohydrate-counting features, enhanced by image recognition and artificial intelligence technology, can significantly improve the accuracy of measuring carbohydrate intake [58].

The majority of participants responded positively to the suggestion of incorporating reminders and notifications into mHealth apps. Reminders and notifications have proven effective in assisting patients in adhering to their medication regimens [59]. A recent study revealed that reminders for diabetes-related appointments, medication adherence, screening, and routine laboratory tests had a substantial impact on the clinical outcomes of the patients [60]. The IDF (International Diabetes Federation) Europe has also recommended the inclusion of SMS text messages or notification features within mHealth apps, recognizing their importance in preventing long-term complications among patients with diabetes [61].

Patient-provider interactions facilitated by digital technology can significantly enhance the quality of care for patients. This includes assistance in coordinating care, providing information about medical conditions and treatment decisions, aiding in disease management, and supporting the learning of health behavior changes [62]. Additionally, wireless communication and integration between various apps and devices are crucial for health data collection, storage, and sharing with health care professionals.

### Limitations of the Study

The recruitment method for participants in this study primarily involved advertising through social media. It is important to note that this recruitment approach may have attracted participants with a higher level of technology literacy. However, it is worth mentioning that even with this bias, 60% (9/15) of the study participants had never used mHealth apps for self-management of diabetes, indicating that a significant portion of the sample had limited prior experience with such technology. While this study used maximum variation sampling to recruit a diverse group of participants, it is noteworthy that a majority of the participants were from urban regions in the Klang Valley. These areas typically have stable internet connectivity and higher levels of technology literacy. As a result, the study findings may not fully represent the experiences and perspectives of the Malaysian population with diabetes residing in rural regions. Additionally, due to constraints in time and resources, snowball sampling was applied to recruit patients with diabetes of Indian ethnicity. This may have introduced some limitations in the diversity of the participant pool. It is worth acknowledging that the snowball sampling method may have introduced certain biases into the study. While it is recognized that this method does not guarantee sample diversity [63], the research team made efforts to promote sample heterogeneity through the primary sampling method, which was purposive sampling during recruitment.

Another limitation of this study was the recruitment of English-speaking patients. Malaysia has a multilingual population, with Malay, Chinese, English, and Tamil being the main spoken languages. Because of resource constraints, the study was limited to recruiting patients who could understand and converse in English. As a result, the study might not have captured the important perceptions of Malaysian individuals who do not primarily speak English, particularly regarding their perceived benefits, needs, and preferences related to using mHealth support. This limitation should be considered when interpreting the study’s findings.

### Implications for Practice and Future Research

The findings of this study indicate that the majority of participants held a positive perception regarding the use of mHealth for self-management of diabetes. Nonetheless, it is crucial to emphasize the importance of education and training to ensure that patients, particularly older individuals, can effectively use mHealth technology for their diabetes management needs. To address the needs of individuals with low technology literacy, it is essential to develop mHealth app designs that are simple and easy to navigate. This user-friendly approach can significantly facilitate the usage of mHealth apps among this population. Furthermore, the study has highlighted various barriers to using mHealth support and has suggested the incorporation of user-centered design features to address these challenges effectively. This emphasis on user-centered design is crucial for meeting the needs of individuals in their daily tasks, ultimately contributing to improved patient health outcomes [64]. Additionally, the study underscores the vital role of health care providers in promoting the use of mHealth among their patients. When health care providers actively encourage patients to engage in self-management at home through mHealth, it is likely that more patients will adopt and benefit from these digital tools.

Future research could extend its focus to include patients from rural areas to provide a more comprehensive understanding of mHealth usage in diverse settings. Additionally, exploring the impact of factors such as behavioral characteristics, health literacy, cultural differences, and socioeconomic disparities on patients’ engagement with mHealth support would offer valuable insights. Furthermore, investigating clinicians’ perceptions regarding the use of mHealth support in patient care could provide a well-rounded perspective on the integration of these technologies into health care practices.

### Conclusions

In summary, this study offers valuable insights into the perspectives of Malaysian patients with diabetes regarding the use of mHealth support for diabetes self-management. The participants’ willingness to embrace mHealth app support was motivated by their recognition of the perceived benefits and recommendations from health care providers. These perceived benefits encompassed the ability to track and monitor diabetes control, aid in lifestyle modifications, and facilitate more informed treatment decision-making for health care professionals. Significant barriers to the adoption of mHealth app support are a lack of awareness about the availability of mHealth apps, insufficient recommendations and information on using these apps from health care providers, limited digital literacy among users, and apps that may not align with the specific needs of individual users. The study has also shed light on the patients’ requirements for future mHealth apps, emphasizing the importance of incorporating engagement features, user-friendly designs, educational information, and carbohydrate-counting functionality in these apps.

## Appendix

Multimedia Appendix 1 COREQ (Consolidated Criteria for Reporting Qualitative Research) checklist.

Multimedia Appendix 2 Interview guide.

## Abbreviations

COREQ: Consolidated Criteria for Reporting Qualitative Research
HbA_1c_: hemoglobin A_1c_
HITAM: Health Information Technology Acceptance Model
IDF: International Diabetes Federation
MARS: Mobile Application Rating Scale
mHealth: mobile health
TAM: Technology Acceptance Model
