# Supplementary material for: Perspectives and Needs of Malaysian Patients With Diabetes for a Mobile Health App Support on Self-Management of Diabetes: Qualitative Study
Source: JMIR Diabetes. 2023 Oct 23;8:e40968. doi: 10.2196/40968 (PMC10628693; doi:10.2196/40968)
Supplement: Multimedia Appendix 2 [file diabetes_v8i1e40968_app2.docx]

**Appendix I: Interview guide**

| Question | Elaboration questions |
| --- | --- |
| Which health app(s) have you used before? | Do you still use that/those app(s)? (If multiple apps) Which of those apps are still on your device? Which of these do you still use? Which one(s) would you like to talk about today? |
| Have you encounter any challenges when you are using the health apps? | Is it too complicated to be used or anything else? |
| For approximately how long have you used (did you use) this app? | How often do/did you use it? (If discontinued) Why did you stop using the app? |
| What do/did you like about this app? | Does/did the app fulfil your needs? Why or why not? Do/did you enjoy sessions with your health app? |
| Have you encountered any challenges when you are using the health app? | Further prompts: Is it too complicated to be used or anything else? |
| (If partcipants never used any health-related app) What are your main reasons for not using health related app? | Do you have concerns about using an app for health purposes?  Further prompts: Is it something related to cost or whether it's too difficult to use. |
| Would you ever consider using an app to help manage your diabetes? | Why or why not? |
| What type of feature/information would you want in the app to make it useful for diabetes management? | May prompt: Education, reminders to check blood sugars, recipes/diet info, exercise, medication, nutrition information, general info about diabetes, stress and psychological health |
| How does your current practitioner encourage you to monitor your diabetes? | Has your practitioner ever mentioned/recommended a health monitoring app to you? |
| Do you regularly monitor blood sugar levels? | What methods do you use to keep track of your sugar levels? |
| What techniques, if any, do you use to ensure you always remember to take your medications? | Do you ever forget?  Would an app that reminded you to take your medication be helpful? |
| Do you have any alternative methods to motivate yourself to eat healthy and exercise regularly? | If so, please describe them. |
| How do you currently access information if you want to educate yourself about diabetes? | What are the issues you find with current online diabetes education platforms? |
| What form of information would you find most useful in a health app for diabetes? | Prompts: videos, reading articles, talking to others  What are the perceived benefits and barriers to using these different forms |
| Do you think receiving daily text messages or emails with reliable diabetes information would be useful for you? | If not, how often would you like to receive information? |
| Do you think that your skills or education impact your ability to use digital technology for your diabetes? | Why? Explain |
| What is your view of information stored on the cloud/internet? | Do you have concerns about privacy? |
| Do you think that using an diabetes related health app will help you to better manage your diabetes? | Why? Explain |
